# Maximizing the Application of RAP in Asphalt Concrete Pavements and Its Long-Term Performance: A Review

**DOI:** 10.3390/polym14214736

**Published:** 2022-11-04

**Authors:** Jialin Zhang, Taiwo Sesay, Qinglong You, Hongjun Jing

**Affiliations:** 1 Jiangxi Provincial Communications Investment Group Co., Ltd., Nanchang 330025, China; 2 School of Highway, Chang’an University, Xi’an 710064, China; 3 College of Architecture and Civil Engineering, Xi’an University of Science and Technology, Xi’an 710054, China

**Keywords:** reclaimed asphalt pavement, pavement preventive maintenance treatment, RAP repeated recycling, long-term performance

## Abstract

The use of recycled asphalt pavement (RAP) materials in asphalt concrete pavements (ACP) brings significant cost and environmental benefits. In practice, however, the amount of RAP readily available far exceeds the amount being utilized in ACPs, which still leaves the problem of excess RAP in the environment partially solved. Additionally, ACPs containing RAP materials (i.e., RAP-ACPs) can still be landfilled after they have reached the end of their useful life, which may restore the original environmental waste problem. To address these, researchers have demonstrated different ways to maximize the application of RAP in ACPs. Among them, the use of RAP in pavement preventive maintenance (PPM) treatments and the repeated recycling of RAP-ACPs (i.e., R^n^AP) are specifically discussed in this review. It is envisaged that, by promoting these two practices, the application and benefits of RAP can be further maximized to improve sustainability. This review also discusses the long-term behavior of RAP-ACP, which is crucial to inspire confidence in the wider application of RAP in ACP. Studies on RAP-PPM have shown that virgin PPM treatments can successfully accommodate RAP materials by adjusting their mix design. So far, research on R^n^AP has been limited to how multiple-recycling affects the performance properties of the blends, showing improvements in rutting resistance and moisture susceptibility but little effect on linear viscoelasticity and cracking. Overall, the lack of sufficient research is considered to be the biggest challenge in facilitating the implementation of these two sustainable RAP technologies. Little or nothing is known about the bonding mechanisms between RAP and fresh PPM binders, the molecular and chemical changes in R^n^AP binders, or the functional performance characteristics, actual pavement performance, and long-term performance of both RAP-PPM and R^n^AP blends. An understanding of these aspects is very relevant to maximize and continue the beneficial reuse of RAP in ACPs while safeguarding human and environmental health.

## 1. Introduction

Asphalt concrete pavements (ACP) are designed and built to last for a limited period of time, usually between 15 and 35 years. After this period, if the surface layer cannot be repaired, it must be completely removed and replaced. One way of disposing old ACP materials is to dispose of them at a designated landfill or dump site. Landfill disposal is not a sustainable option for obvious reasons, such as environmental concerns and cost issues related to equipment and energy costs. For many years, transport agencies, contractors, engineers, and pavement researchers have shown keen interest in the ACP recycling option because of its many benefits. Firstly, ACP recycling can save significant amounts of money. The National Asphalt Paving Association estimates that the use of recycled pavements in infrastructure could save nearly $2 billion annually [1]. A major factor in this cost reduction is that ACP recycling uses fewer natural resources, such as stone, aggregate, and asphalt [2]. Secondly, ACP recycling reduces landfill disposal, which is beneficial to the environment [3,4]. In addition, greenhouse gas (GHG) emissions are reduced through ACP recycling, as less virgin asphalt binder is involved [2]. Performance-wise, previous studies have reported that the final product from ACP recycling is durable. It has better stiffness, which reduces the probability of rutting damage, leading to fewer repairs and maintenance in the future [1]. However, the effects on fatigue resistance and low-temperature cracking performance are conflicting [5,6,7,8,9,10].

ACP can be recycled by extracting the asphalt binder or aggregate fraction from the old ACP and adding it to the newly produced asphalt mix in a specified quantity, or by incorporating the RAP directly into new asphalt mixtures as it is without separating the binder from the aggregates. The recycled asphalt binder is referred to as the RAP binder and the recycled aggregate is referred to as RAP aggregate. Traditionally, RAP is incorporated into hot- or warm-mix asphalt in amounts no more than 40% [2], thus leaving behind a surplus in the RAP stockpile that largely remains unutilized. Several efforts have been made to fully exploit RAP in asphalt pavements. Recent studies have shown that it is possible to maximize the use of RAP in ACP with the help of rejuvenating and softening agents, increasing the RAP content to 100% [11]. However, various concerns have been expressed about the effects of high RAP amounts on mechanical properties such as fatigue cracking, low-temperature cracking, and moisture susceptibility [4,12,13,14,15,16,17,18]. In addition, it is not clear how the use of high amounts of RAP can impact the long-term performance of asphalt pavements. Nowadays, alternative options such as RAP application in pavement foundation layers (base and sub-base) is also being considered because it can consume higher volumes of RAP materials [19]. Improvements in recycling construction techniques such as hot in-place recycling (HIR) and cold in-place recycling methods are also very promising to maximize the application of RAP in ACP [20,21,22,23].

Multiple published reviews on RAP have extensively discussed the above-mentioned approaches to give a wholistic understanding of their current state of practice [2,11,24,25,26,27,28,29,30,31,32]. However, the literature lacks an in-depth investigation on three other important topics including the application of RAP in PPM treatments, RAP repeated recycling, and RAP-ACP long-term performance, which are all very relevant to maximizing and extending RAP usage in ACPs. Therefore, the objective of this review was to provide sufficient context on the existing state of knowledge on these topics in order to establish a general trend in the various research findings, identify potential challenges, and provide some perspectives for future research.

Following this introduction, Section 2 presents a brief overview on the application of RAP in ACPs. Although this section is not the primary focus of this review, it serves as a background for the main topics discussed. Section 3 discusses the existing state of knowledge on RAP-PPM and R^n^AP and summarizes the general trend in the research findings. The long-term behaviors of RAP-ACP are discussed in Section 4 in terms of structural and functional performance as well as influencing factors. Section 5 reflects on the literature review, identifies some potential challenges, and provides some recommendations for future research. Finally, the summary and conclusions of the study are presented in Section 6.

## 2. Brief Overview on the Application RAP in Different Asphalt Pavement Layers

**Figure 1:**
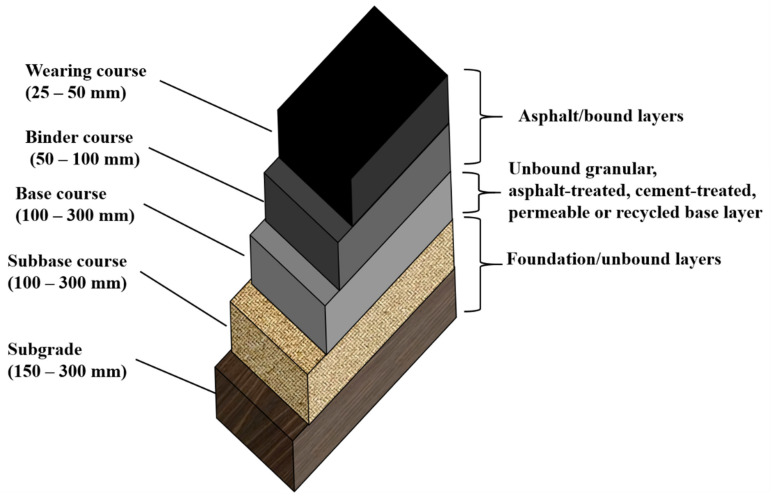
Different layers of typical asphalt concrete pavement.

Asphalt pavement is made up of multiple layers, namely: subgrade and sub-base, which constitute the foundation or unbound layers; the base, which can be unbound granular, asphalt-treated, cement-treated, permeable, or recycled [33]; and the asphalt layers at the surface, as shown in Figure 1.

A very important part of the ACP design process is the selection of appropriate pavement materials for the construction of the individual layers. These materials show a complex response when subjected to changes in load, temperature, and moisture. The engineering performance of paving materials depends on the relative composition of their constituents. In order to maximize the benefits of RAP, the transportation departments of several countries around the world—including in China [34], Japan [35], South Africa [24], USA [36,37], Europe [38], Australia, Victoria, New South Wales, Tasmania, and New Zealand [39]—allow its use in the various layers of ACP. The following subsections provide further details on the use of RAP in different ACP layers.

### 2.1. RAP Usage in the Asphalt Surface/Bound Layers

The surface layer is the upper layer of the ACP and provides smooth, durable, and wear-resistant characteristics while retaining sufficient friction for road safety. It should also be resistant to water-induced damage, and must be strong enough not to rut under the action of traffic.

The application of RAP material in ACP surfaces has been the subject of intensive research due to its environmental and economic benefits. The greatest use of RAP is in hot-mix asphalt (HMA). It involves mixing RAP with new or ‘virgin’ aggregates, base asphalt binders, and/or rejuvenator in a central hot-mix plant to produce a recycled asphalt mix. The percentage of RAP allowed in surface mixes typically ranges from less than 20% to more than 50%, depending on the agency or country user guidelines.

In China, the utilization of RAP in surface courses is not very common, despite its significant economic and environmental benefits [40]. Nie [34] conducted a laboratory study to evaluate the performance of asphalt mixes in terms of fatigue resistance, moisture sensitivity, high-temperature stability and low-temperature cracking. All RAP mixes met the performance requirements, except for 30% of the RAP mixes that failed in terms of moisture resistance and low-temperature cracking. The study was from a research project in China where different concentrations of RAP (0%, 10%, 20%, and 30%) were used in the surface mixes. A 20% RAP content was determined to be the best performance.

Japan’s road sector was already an example of an effective RAP recycling system in 2013. After two years, 99% of the total RAP was reused [35]. A small amount of RAP was used in the base layer, while the majority was added to the new HMA-WMA mixture, introducing an average of about 47% by weight of RAP [35].

The South African practice allows up to 40% RAP in ACP surface layers [24], while in Australia, Victoria, New South Wales, Tasmania, and New Zealand allow RAP contents of at least 15% [39]. Higher amounts are possible if contractors can demonstrate that they are using appropriate plant technology and quality control procedures [39]. Other parts of the country, such as Queensland, South Australia, and Western Australia, do not allow the use of RAP for road surfaces [39].

In the United States of America (USA), a threshold RAP amount of 30% is recommended for better structural and functional performance of asphalt surface mixtures [36,37]. The Federal Highway Administration (FHWA) defines asphalt concrete containing more than 25% RAP (of the total mix weight) as a high RAP content mix [41]. However, agencies are reluctant to use high RAP content in ACP surface layers due to the drawbacks associated with high RAP. Currently, the addition of limited amounts of aging RAP binder to fresh HMA increases the stiffness of the mixture [4,16], which subsequently leads to various forms of cracking such as fatigue cracking and low-temperature cracking. By further increasing the amount of RAP, this problem only becomes more severe. Nevertheless, there are many non-negligible benefits to using high RAP in the surface layer, such as improved resistance to rutting [42,43] and water-induced damage [42], as well as cost and environmental benefits [2].

Research has shown that, with certain modifications, it is possible to maximize the application of RAP in ACP surface layers without compromising performance. Common techniques that allow the use of high RAP are the use of soft binders, the addition of rejuvenators or softening agents, or the use of WMA additives [2,26,28,30,44]. Softening agents usually reduce the viscosity of the aged binder, while rejuvenators help facilitate the rebalancing of the chemical composition in the aged binder that has lost its light molecular weight fraction during construction and use. In any case, the type and quantity of the selected additive should be carefully selected and its effectiveness evaluated by binder and mixture tests [30].

### 2.2. RAP Usage in the Base and Sub-Base Layers

The typical base layers can be unbound granular, asphalt-treated, cement-treated, permeable, and recycled [33]. The sub-base is typically located at the bottom of the base course layer and laid on top of the subgrade, serving to distribute the load over the subgrade [33]. However, the sub-base is not usually used unless heavy traffic is involved, or if the subgrade is very weak [33].

Most studies on the application of RAP in ACP have focused on asphalt layers, ignoring the potential of unbound layers (base and sub-base). However, evidences have shown that RAP can also be used in base and sub-base mixtures. The base layer is thicker than the asphalt layer; therefore, more recycled material can be consumed, thus reducing the amount of RAP in stockpiles. Cost-wise, research has shown that by partially replacing virgin aggregates with RAP aggregate in the base and sub-base, material cost savings of approximately 30% can be achieved [45].

In addition to the economic and environmental benefits, RAP significantly influences the structural behavior and performance of the unbound layers. The modulus of elasticity (M_R_), an important parameter in the Mechanistic-Empirical Pavement Design Guide (MEPDG), reflects the potential to resist permanent deformation. For typical unbound aggregates, the modulus of elasticity ranges between 125 MPa and 300 MPa [46]. However, previous researchers have noted that RAP aggregates tend to have a higher modulus of elasticity than conventional unbound aggregates [47], which, in turn, can improve rutting resistance. This effect on the mechanical properties and performance of unbound layers is clearly influenced by the amount of RAP in the mixture [48,49]. When used as an unbound aggregate, studies have shown that 100% RAP reduces strength and increases the potential for permanent deformation of the unbound base [50]. Plati and Cliati [51] reported that 100% RAP material and 50% RAP/50% virgin aggregate (VA) material produced M_R_ results similar to those investigated for VA, particularly at increased compaction pressures. Others systematically agree that RAP addition to VA (i) reduces dry density [52], (ii) reduces bearing capacity (as measured by the California Bearing Ratio) [52], and (iii) increases the modulus of elasticity [47,53,54,55]. However, the question usually arises as to how to utilize the high RAP amount in the base layer without diminishing the mechanical properties.

It has been shown that stabilized granular materials can allow for high RAP amounts. Mechanical (compaction, geotextiles), chemical, and cementitious (including hydraulic, zeolite, and asphalt cement) stabilization methods are all potentially viable methods, depending on the characteristics of the RAP material and the requirements of the end-use [19,55,56] 

## 3. State of the Art

### 3.1. Pavement Preventive Maintenance Treatments Using RAP (RAP-PPM)

**Figure 2:**
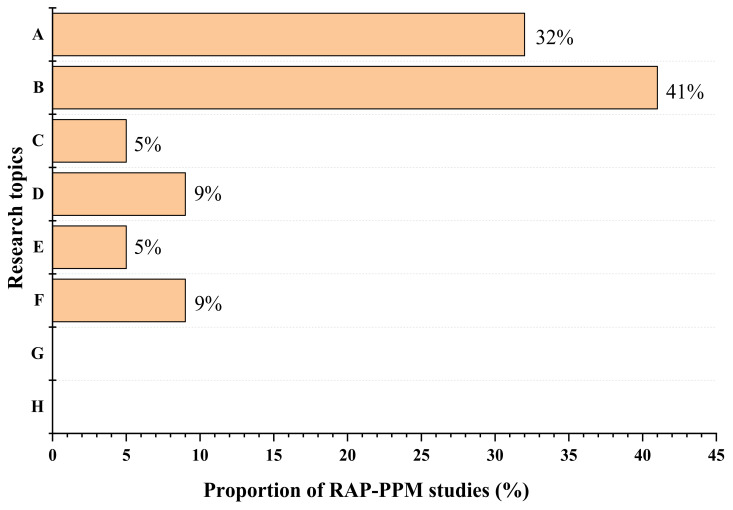
Current trend in RAP-PPM research. A = Modifications to accommodate RAP in PPM treatments (e.g., mix design criteria, additives characteristics, mix design tests, etc.). B = RAP-PPM mixture performance (e.g., rutting, cracking, moisture susceptibility, workability). C = Adhesion of particles to new binders. D = Functional performance characteristics (skid resistance, texture, surface friction, noise, etc.). E = Availability of actual pavement performance data. F = Cost-effectiveness of RAP-PPM treatments. G = Long-term performance of RAP-PPM surfaces. H = Interaction behavior between RAP asphalt and fresh PPM binders.

Despite the many years of RAP application to different layers of asphalt pavements, significant amounts of surplus RAP still remains in stockpiles or landfills, which has negative impacts on the environment. Nowadays, the extent of the asphalt road network has increased, leading to huge funding requirements for carrying out activities for pavement maintenance, rehabilitation, or reconstruction. Recently, innovative uses of RAP in pavement preventive maintenance (PPM) treatments have been considered as a very sustainable approach to dealing with the two-fold problems of surplus RAP and the high costs of pavement maintenance projects. In order to understand the state of the science on RAP-PPM, Figure 2 provides an overview of current research trends on the subject, presented by focus topic.

In order to successfully incorporate RAP into PPM without compromising performance, it is required to make slight adjustments to the mix design of the PPM treatment mixture. So, in these early stages of RAP-PPM research, the greater percentage of studies have focused on how such modifications can be made (32%), as well as how RAP inclusion will impact the overall mixture’s performance properties (41%), as shown in Figure 2.

The information collected from the studies on RAP-PPM mixture performance are reported in Table 1 in order to observe and highlight the general trends of their findings.

As shown in Table 1, existing data from published literature have so far reported RAP application in four different PPM treatments, including thin hot-mix asphalt overlay (THMAO), micro-surfacing, chip seal, and slurry seal. Regardless of the PPM treatment type, resistance against rutting and moisture damage are significantly improved, while cohesion shows negative results. Research on cracking, workability, and skid resistance were also conducted in few studies with varying outcomes, as shown in Table 1. The following sub-paragraphs discuss in detail the current state of knowledge for each RAP-PPM type.

#### 3.1.1. Thin Hot-Mix Asphalt Overlay (THMAO)

A thin hot-mix asphalt overlay (THMAO) is a dense-graded asphalt mixture designed with small nominal maximum aggregate size (NMAS) of less than 12.5 mm and placed at a thickness below 4 cm using conventional asphalt production and placement operations [64]. The small NMAS of THMAO distinguishes it from other specialty asphalt mixtures occasionally used as thin overlays, such as open-graded friction course (OGFC), stone matrix asphalt (SMA), and ultra-thin bonded bituminous surface (UBBS), which are designed with NMAS of 12.5 mm or above. Despite sharing a design focus on material quality (high-quality aggregates, high-performance asphalt binder, and less natural sand), THMAO does not provide as much structural support as a conventional overlay and should not be used to correct structural defects [64]. Obvious areas of cracking and unevenness are often reflected by a thin overlay. Before the placement of THMAO, it is important to first inspect the existing pavement surface to determine which areas may require additional repair prior to overlay.

**Figure 3:**
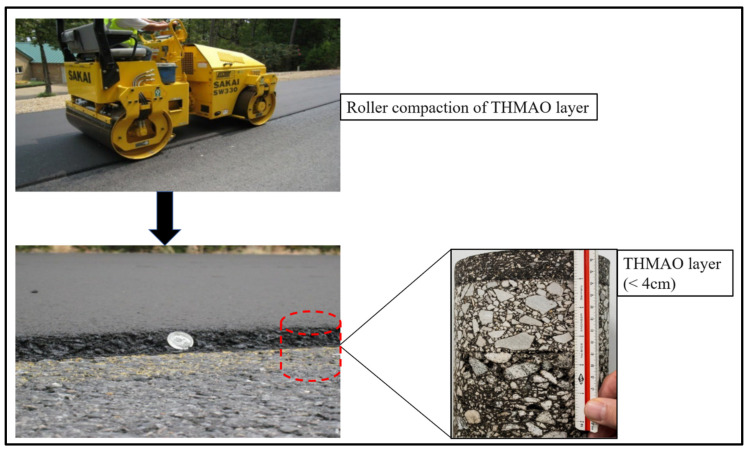
THMAO technology.

The construction of THMAO requires the same equipment as the traditional overlays previously mentioned. However, depending on the mix and rolling pattern, compaction may be achieved with fewer rollers [64]. Figure 3 shows roller compaction of a THMAO layer.

Placement should be done during mild, dry conditions, with a minimum surface temperature of 4.5 °C [64]. A tack coat should be applied after sweeping the surface, with the mix placed immediately after the tack “breaks”. Compaction is critical for smoothness and performance, and rollers should operate as closely as possible to the paver. Thinner lifts may cool more quickly, shortening the compaction temperature window.

Currently, THMAO is considered the primary PPM technology for designing maintenance programs because of its significant advantages in restoring the surface condition and functional performance of pavements [65,66,67]. However, its implementation consumes more natural resources—especially natural aggregates, asphalt, and modified bitumen—which is neither cost-effective nor environmentally friendly. To offset the cost of virgin materials and the use of polymers in asphalt binders, a high amount of RAP has been recommended for use in the THMAO mix designs [65,68]. However, fractionating high amounts of RAP for THMAO application produces more material that passes through the No. 200 sieve [68]. Therefore, in order to fully exploit this resource, the finer-grade proportion of the processed RAP should be mostly used. As previously mentioned, THMAO blends with 4.75 mm NMAS are used for PPM treatments and are typically between 1 and 3 cm thick, which provides the opportunity for consuming more fine-grade RAP [67].

These things considered, fine-graded mixes generally improve the quality of THMAO in many ways; for example, by making it extremely dense and impermeable, while being highly flexible and crack resistant [69]. According to a recent laboratory study by Podolsky et al. [63], it was shown that a large amount of fine-graded RAP (40%) and a regenerating agent (>8% of the total mix) can improve the resistance of THMAO mixes to low-temperature fracture, fatigue cracking, and rutting damage. Other properties of THMAO mixes, including their stiffness and resistance to reflection cracking, have also been reported to be improved by the addition of large amounts of RAP [66]. However, although the increase in THMAO stiffness favored moisture and rutting resistance, the workability of the mixture was adversely affected. Without reducing the large amount of RAP in the THMAO mix, the addition of WMA additives is recommended to address the associated workability issues [42].

#### 3.1.2. RAP in Micro-Surfacing

Micro-surfacing is one of the most advanced pavement surface treatments, remedying a wide range of pavement defects and lasting longer than other surface treatments. This mixture can be effectively used for preventive and corrective maintenance, mainly for restoring skid resistance, filling ruts to restore transverse surface profiles, and repairing weathering and raveling [62]. The successful performance of micro-surfacing in pavement maintenance treatments has led to considerable interest in its application, which can be further developed through the use of RAP materials.

**Figure 4:**
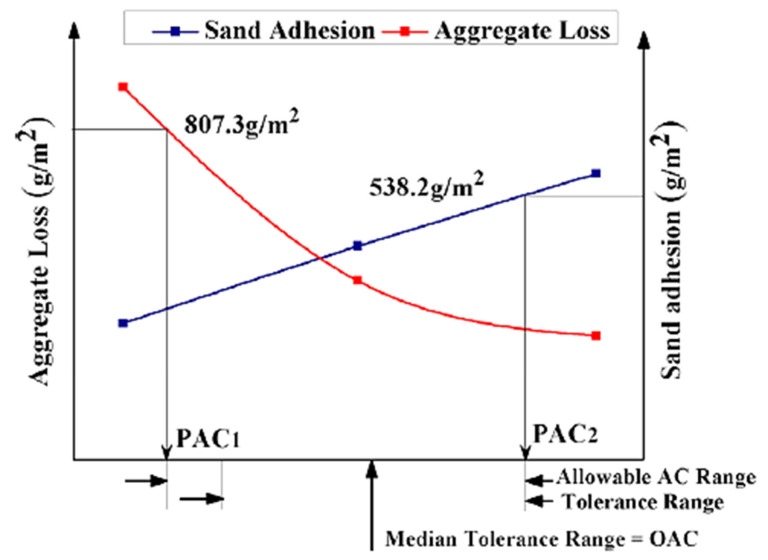
Determination of OAC from ISSA guidelines (Reprinted with permission from Wang et al. [62]. 2019, Elsevier).

Several researchers have noted that RAP can completely or partially replace virgin aggregates (VA) in micro-surface mixtures [58,59,60,62]. The current method for designing mixtures for micro-surfacing generally follows the International Slurry Surfacing Association (ISSA) TB111 standard. This uses aggregate loss from the 1 h wet track abrasion test (WTAT) and sand adhesion from the load wheel test (LWT) to determine the lower and upper limits of potential asphalt content (PAC_1_ and PAC_2_, respectively), as shown in Figure 4 [70,71].

**Figure 5:**
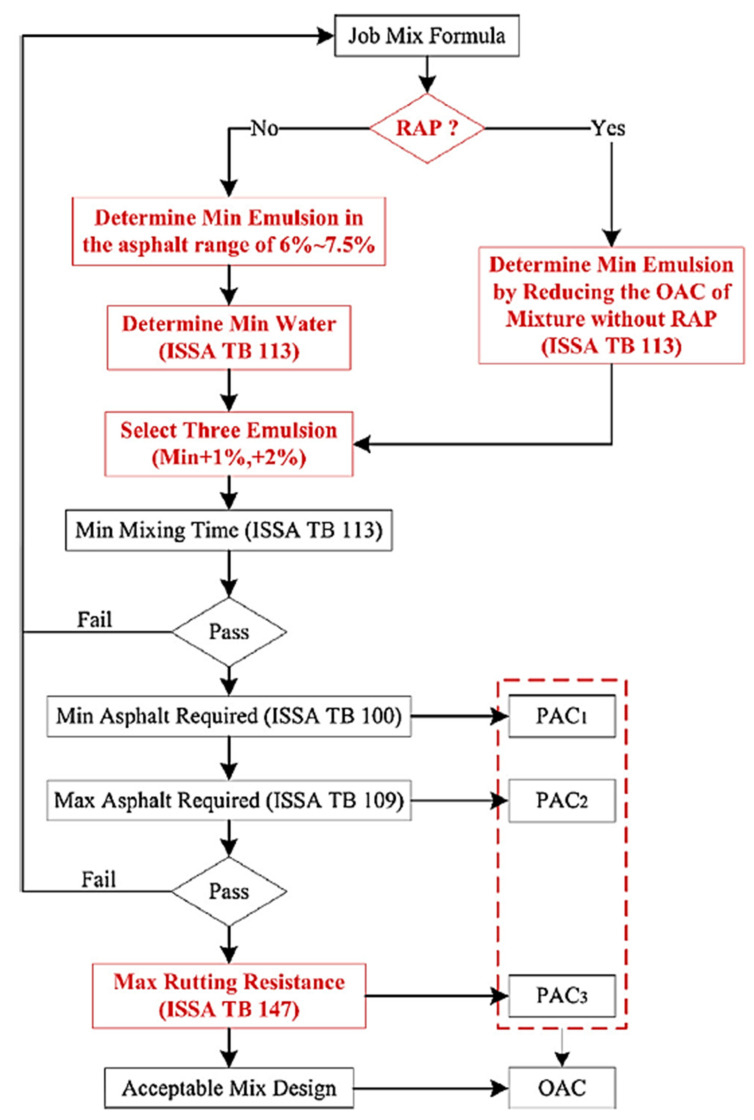
Modified mix design for RAP microstructures (Reprinted with permission from Wang et al. [62]. 2019, Elsevier).

The tolerance limits shown in Figure 4 were provided by the contractor. The optimum asphalt content (OAC) is the median of the tolerance range, and is subsequently adjusted according to engineering judgment to account for expected traffic volumes [72]. This process requires an experienced designer; the choice of OAC is empirical [73]. Recently, Wang et al. [62] proposed a modification to the ISSA micro-surfacing mix design to accommodate the use of RAP, as shown in Figure 5.

As shown in Figure 5, the modified mix design was accomplished by four tasks: (i) three test formulations are formed; the first is a base emulsion for Class III micro-surfacing with conventional asphalt content (6.0–7.5%), with an increase of 1% and 2% to form the second and third formulations, respectively; (ii) PAC_1_ and PAC_2_ are selected according to Figure 4; (iii) PAC_3_ is determined by load wheel testing (LWT) at minimum percentage vertical displacement (PVD) and percentage lateral displacement (PLD); (iv) the OAC is determined by comparing PAC_3_ with PAC_1_ and PAC_2_. If RAP is to be used, the three test equations should be determined by proportionally reducing the OAC of the 0% RAP micro-surfacing mixture. Using the proposed modified mix design of the micro-surface, Wang et al. [62] noted that the addition of RAP influenced the mix design parameters; the OAC decreased as the amount of RAP increased, while the mixing time increased and the mix consistency was improved. There was also a positive impact on the performance of the micro-surfacing mixture, including improved moisture and freeze–thaw resistance, improved skid resistance, and improved rutting resistance.

Robati et al. [58] investigated the effect of using different amounts of RAP on the mechanical properties of micro-surfacing mixtures. It was found that, although the increase in RAP content (0–100%) reduced the cohesion of the mixture and increased the lateral and longitudinal displacements, it still met the minimum requirements set by the International Slurry Spreading Association (ISSA). At the same time, the effects on abrasion and susceptibility to wetting were not significant [58]. A similar study conducted by Poursoltani and Hesami [60] also reported that a micro-surfacing mixture containing RAP met ISSA requirements for cohesion, deformation, and performance in wet environments

In 2010, contractors for Los Angeles County (LAC) completed a project using RAP aggregates in a micro-surfacing mixture and reported positive results [74]. According to the LAC, the residual asphalt content requirements for RAP micro-surfacing were higher than those for VA, while the mix requirements for RAP micro-surfacing, such as mixing time and WTAT loss results, were the same as those for VA micro-surfacing.

Poursoltani and Hesami [60], after evaluating different mix design formulations to accommodate RAP in micro-surfacing mixtures, recommended a minimum asphalt emulsion for 69% RAP to be a maximum of 1% more than a mix with 100% VA. The higher asphalt emulsion for mixes containing RAP is due to the higher surface area of RAP aggregates compared to VA, which thus requires more water addition and relatively lower amounts of additives.

Overall, the above literature suggests that, with the right application, high levels of RAP can be added to micro-surfacing mixtures with both environmental and engineering benefits.

#### 3.1.3. RAP in Chip Seal

Chip sealing is a thin surface treatment consisting of a sprayed asphalt emulsion followed by an aggregate layer. It seals minor cracks and waterproofs existing pavements, on top of which the sealer is applied. Igneous rocks, metamorphic rocks, sedimentary rocks, and manufactured aggregates have all been used successfully for sealing. The use of RAP in chip seals is very economical. However, it is a very new concept and is not widely used. Tarefdar and Ahmad [75] conducted a study in New Mexico to compare the cost-effectiveness of using RAP and VA in chip seals. Three of the six districts selected for the study used RAP seals and VA on their pavements. In District 1, the use of RAP seals was 23% more cost-effective than the use of native chip seals. For District 6, sealing with milling material was 37% more cost-effective than sealing with virgin chips. As a result, there is increasing interest in the use of RAP in sealers.

Transportation agencies looking to maximize the benefits of RAP by extending it into sealer typically test RAP chip seals against many of the criteria traditionally applied to virgin seals to approve the source aggregate and emulsion type. These tests and criteria include compatibility testing, seal grading and sizing, cleanliness and sand equivalents, requirements for the absence of hazardous materials, wear testing, methods for determining emulsion and aggregate application rates, and emulsion requirements and grade selection (e.g., polymer modification, tire rubber modification) [76]. RAP can replace virgin aggregate if the RAP stockpile is fractionated to separate coarse particles for chip sealing and fine particles for micro-surface or slurry sealing. The RAP stockpile must not have metal, fiber, or soil contamination. In addition, the performance of the source aggregate must meet additional requirements based on the type of treatment in order to have performance comparable to that of the virgin treatment [76].

Rahaman et al. [57] extracted RAP materials from an ultra-thin bonded bituminous surface (UBBS) and evaluated their chip retention based on the American Society of Testing and Materials (ASTM) sweep test. Although the results were not encouraging, Rahaman et al. [57] determined that RAP can improve chip retention if the right type of aggregate and asphalt emulsion is available. The University Research Centre (URC) in the USA, a leading institution in the use of RAP in PPM treatment, has conducted regular monitoring to compare the in-service performance of RAP and virgin chip seals. It was observed that there was no significant chip loss in the RAP section, whereas chip loss was observed in the chip test section with pre-applied aggregates. In addition, the RAP section performed equal to or better than the virgin chip seal test section in terms of surface cracking, wheel-path rutting, and surface texture measurements.

For other agencies, such as the New Mexico Department of Transportation (NMDOT) and San Bernardino County, California (SBC), less-than-optimum pavement friction is an issue, especially if the RAP consists of more than just surface mixes, or if the aggregate source for other mixes is not a surface-approved material [76]. Tarefdar and Ahmad [75] observed the surface conditions of the New Mexico Highway 124 (NM124) at different stages of a chip seal project in New Mexico that used 100% RAP. Many loose pieces were observed on the chip seal surface constructed a day earlier. However, the seal constructed a week earlier appeared to be well compacted with very few loose/unbonded fragments. When this pavement was investigated one year after construction, it was observed that there was little surface distortion apart from a few loose and broken aggregates. It is known that chip seals increase surface friction due to the aggregate grade and macrotexture, but the aggregate particles are easily polished depending on their mineral composition [76]. Tarefdar and Ahmad [75] also observed the chip seal surface to be very dark, indicating a low level of oxidation. The SBC successfully applied a mist seal to the chip surface to reduce chip loss and to uniformly color the pavement surface black. Other local projects have used polymer-modified recycled emulsions (PMREs) in RAP seals to improve performance [74]. Chip seals consisting of RAP aggregates and PG 76-22 tire-rubber-modified asphalt binder were also used in 2013 in the Lake Los Angeles area. The purpose of this project was to determine if the non-preheated 0.375” and 0.3125” RAP aggregates were as compatible as the original aggregate. The chip seal performed positively despite construction issues such as variable/high moisture content, RAP aggregate cleanliness on the first day of the project, and unfavorable weather conditions [74].

Overall, the use of RAP in chip seal applications is a sustainable and cost-effective practice, but some changes to the application rates of chip seal emulsions and RAP treatments are required [76].

#### 3.1.4. RAP in Slurry Seal

Slurry sealing is a commonly used emulsion-based pavement maintenance method that is environmentally friendly and easy to construct. According to Wang and Wang [77], slurry sealing improves pavement surface friction more than chip sealing, THMAO, or crack sealing. A slurry seal is a mixture of aggregate, asphalt emulsion, water, and additives that is mixed and spread on a prepared surface. The use of RAP materials (RAP slurry) in slurry sealing is a relatively new development and is becoming more common. Recently, researchers have investigated the effect of RAP on the performance of slurry sealing mixtures.

Saghafi et al. [61] replaced 87.5% of the total aggregate in the slurry seal mixture with RAP material and found that the RAP slurry seal mixture required more water than a slurry seal mixture containing 100% VA. In addition, the RAP slurry seal mixture required 19% less asphalt binder than the original slurry seal, which meant that it cost 14% less overall than the original slurry seal mixture. The residual asphalt on the RAP aggregate contributes to the asphalt content; thus, the virgin residual asphalt content requirement is lower [74]. The emulsified bitumen used in slurry seals can be slow-setting or fast-setting. Fillers, such as cement and slaked lime (0–3%) depending on the dry aggregate weight of the mixture, are used in slurry seals to increase the consistency of the mixture and to adjust its setting and curing time. Saghafi et al. [61] observed that, although cement or slaked lime reduced the setting time of VA slurry seals by approximately 30 min, they did not show any significant change.

Meanwhile, the effect of RAP incorporation on the performance of slurry seal mixtures is still being investigated. Recently, Ye [78] tested the wear resistance, crack resistance, and rutting resistance of slurry seal mixtures containing 100% RAP and VA. Based on the results of the Hamburg Wheel Tracking (HWT) installation, it was observed that slurry seals made with 100% RAP generally performed as well as or better than those made with VA. Saghafi et al. [61] also showed that RAP slurry seal mixtures outperformed VA slurry seals in terms of abrasion resistance and smaller lateral displacements.

The limited available literature on RAP slurry seals suggests that it is a very promising technology for pavement maintenance and should be further investigated. The specifications for the use of RAP slurry seals will continue to be refined through the knowledge gained from the performance of completed projects.

### 3.2. Repeated Recycling of RAP (R^n^AP)

The application of RAP materials in ACP is a well-established practice, as previously discussed. The resulting RAP-ACP is also expected to go through the same pavement life-cycle as any conventional ACP that has new materials. As a consequence, it is expected to serve for a given period of time before undergoing major maintenance or rehabilitation processes. A necessary question arises as to whether the severely aged materials from RAP-ACP can be reused again in another round of pavement recycling. If, however, the ACP materials are considered useful for recycling only once, the removed RAP-ACP materials will be disposed again in landfills or stockpiles at the end of the RAP-ACP service life. In a way, this is a postponement of the environmental problems the solution of which was attempted by the first round of recycling. Therefore, it is necessary to investigate the potential of ACP to be recycled more than once.

**Figure 6:**
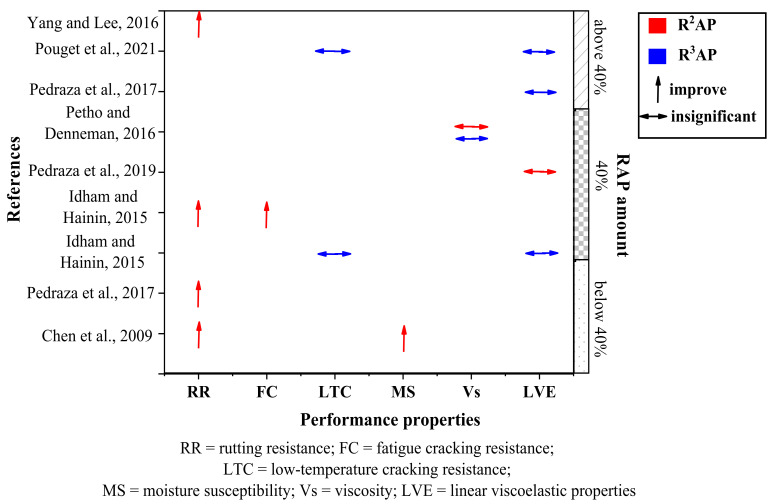
Effect of repeated recycling on mixture properties. Yang and Lee [86]; Pouget et al. [85]; Pedraza et al. [82]; Petho and Denneman [84]; Pedraza et al. [83]; Idham and Hainin [81]; Pedraza et al. [82]; Chen et al. [79].

In recent years, there has been a growing amount of attention paid to this aspect of RAP research because it presents an excellent opportunity for maximizing the cost and environmental benefits of RAP. There has been specific interest in how multiple recycling affects the performance properties of the resultant R^n^AP mixture. Through an eco-mixture formulation, previous studies [79,80,81,82,83,84,85,86] have shown that ACP can be subjected to several rounds of recycling without losing its properties. So far, attempts have been made to recycle ACP to a maximum of three times (R^3^AP), and the effects on properties including rutting resistance, fatigue cracking, water durability, viscosity, linear viscoelasticity, and low-temperature cracking were evaluated. The results are summarized below in Figure 6.

According to Figure 6, subjecting ACP to two or three rounds of recycling may not have an overall detrimental effect on mixture properties. The most crucial characteristic of RAP material that would seriously affect the properties and performance of R^n^AP mixes is aging of its binder. Aging of binder occurs during construction as well as the service-life of asphalt pavements through some major mechanisms such as oxidation, volatilization, exudation, and separation [87]. The viscosity and LVE properties (complex shear modulus and phase angle) are the most vital properties in the design of recycled HMA, which have been reported to be greatly influenced by aging [87].

Petho and Denneman [84] prepared asphalt mixes containing multiple-recycled RAP in the laboratory and studied the viscosity behavior. After the first RTFO aging, an increase in binder viscosity was observed. After the subsequent second and third rounds of RTFO aging, the viscosity of the R^n^AP binders did not show any significant increase. Conversely, Lee [88] reported that the viscosity of R^n^AP binders could be very high and would require a softening agent or rejuvenator to lower it to its target value. However, one major challenge faced is in determining the optimum rejuvenator dosage for R^n^AP binders. For typical RAP binders, the mixing chart developed by the Asphalt Institute (AI) is used by some countries such as Taiwan to estimate this amount [86]. However, for R^n^AP binders which can have very high viscosities, the dosage of the recycled agent may be over-estimated by the AI chart, resulting in softer-than-expected recycled R^n^AP binders [86]. Yang and Lee [86] proposed a modification to the AI chart that can be used to determine the rejuvenator dosage for specifically achieving the target viscosity of an R^n^AP binder.

In addition to viscosity, the study of rheology is one of the methods most commonly used in the binder characterization and its use is present in most of the studies on bitumen aging. During the multiple aging processes of R^n^AP binder, some of the components can be lost and the rheological behavior of the R^n^AP binder will consequently differ from the virgin binder. The general conclusion from previous studies is that aging increases the complex modulus and decreases the phase angle, thereby making the binder stiff [87]. Although stiff binders produce mixtures with better rutting resistance and moisture resistance, they are usually prone to cracking. As shown in Figure 6, some studies reported that multiple recycling does not significantly affect LVE properties [81,82,83], while others have shown improvements in rutting resistance [79,81,85,86] and fatigue cracking [81]. More recently, Pouget et al. [85] investigated the effect of multiple recycling of RAP on different technical properties of asphalt mixes, including thermomechanical properties (linear viscoelasticity, thermomechanical coupling at low temperatures, and crack extension), moisture resistance, and mix compactness. The study was carried out under the French IMPROVMURE (Innovation in Materials and Processes for Asphalt Mixtures) project, the main objective of which was to determine the effects of continuous recycling at different recycling rates and with different manufacturing techniques (hot and warm processing) over the life-cycle of the pavement material. RAP was added to new asphalt mixes at 40% and 70% under hot- and warm-mix conditions, and the resulting mixes were recycled three times in the laboratory through short- and long-term aging processes. Despite some concerns regarding the effects of compaction and moisture resistance of the mixes, none of the investigated thermomechanical properties decreased after several cycles of HMA recycling.

## 4. Long-Term Behavior of Asphalt Pavements Containing RAP

Although the recycling of RAP can reduce costs, there are still some concerns about ACP containing RAP, particularly in the load-bearing layers. A major concern is its durability and long-term performance under various exposure conditions. Knowledge of the long-term performance of RAP-ACP is important, and without it, confidence in its widespread implementation may be reduced.

### 4.1. Long-Term Structural and Functional Performance of RAP-ACP

Different approaches have been used to study the long-term behavior of RAP-ACP, and include laboratory studies on samples from sections of RAP-ACP that have been in operation for several years, laboratory simulation of long-term aging conditions of RAP mixtures, or by field performance monitoring of RAP-ACPs. Table 2 gives the summary results from studies related to the long-term performance of RAP-ACP as compared to conventional virgin mix ACPs. For each study, details on the performance investigated, the long-term performance duration, the RAP amount used, and the performance evaluation metrics are presented in Table 2.

As can be seen in Table 2, both the long-term structural and functional properties of ACP surfaces are affected by RAP usage. When compared with traditional ACPs, RAP-ACP has worse cracking properties and similar performance in terms of rutting, raveling, and ride quality. In general, in asphalt mixtures containing RAP, the long-term aged RAP binder is at least partially mixed with the new asphalt binder, resulting in a stiffer mixture. Studies have shown that RAP-ACP increases the stiffness of RAP mixes after many years of use, thus causing such mixes to be less prone to rutting [91,95]. However, the main issue is their resistance to different crack types, which become more severe as the amount of RAP increases.

Huang et al. [90] carried out a laboratory study of HMA mixtures extracted from four field projects in which test sections of HMA with different RAP percentages (0, 10, 20, and 30%) were constructed for long-term observation. The asphalt binders used in these projects included PG 64-22 and two styrene–butadiene–styrene (SBS) polymer-modified asphalt binders, PG 70-22 and PG 76-22. The RAP aggregates were sourced from limestone and gravel and screened to pass through a No. 4 sieve (4.75 mm).

After 4 years of use, the test section containing 30% RAP showed slightly more cracking than the test section containing 20% or less RAP. The results of the laboratory study by Huang et al. [90] also verified the field observations. Overall, Huang et al. [90] concluded that the inclusion of 30% RAP in the mix of PG 64-22 conventional asphalt binder significantly affects long-term fatigue cracking performance, which is also consistent with results elsewhere [89,91,98].

By exploring data from the Long-Term Pavement Performance Specific Pavement Study experiment 5 (LTTP SPS-5) program, Gong et al. [98] and Wang [99] showed that the long-term cracking resistance of RAP-ACP could be improved by using a thick overlay. Furthermore, Gong et al. [98] and Wang [99] recommended intensive pre-laying preparation or complete removal of existing failure surfaces prior to the placement of overlays in order to favor fatigue cracking, transverse cracking, and longitudinal cracking performance. According to Dong et al. [89], the cracking mechanism on RAP-ACP surfaces starts at an early stage, but is not so severe as to be beyond repair. Their study was also based on data from the LTTP SPS-5 experimental project and field monitoring, conducted every 2 years for the first 8 years and then annually until repair. They determined the timing of cracking for four types of cracks (alligator, longitudinal wheel track, longitudinal non-wheel track, and transverse cracks), and found that, although early fatigue cracking occurred in the portion of the pavement containing 30% RAP, there was no significant ‘severe’ alligator fatigue damage.

In addition to the effect of RAP on long-term structural performance, Table 2 also presents the results of literature studies related to pavement ride quality. The IRI is generally used to quantify pavement ride quality, and previous studies mentioned in Table 2 and elsewhere [98] have concluded that the surface roughness of RAP-ACPs is similar to that of those without RAP.

### 4.2. Influencing Factors on RAP-ACP Long-Term Performance

Factors ranging from RAP mix design parameters (e.g., RAP amount, gradation, moisture content, asphalt emulsion content, and rejuvenator dosage), construction processes, or the influence of warm-mix asphalt additives are known to individually or collectively influence the long-term performance of RAP-ACP.

Zhu et al. [100] designed three different gradations of asphalt emulsion cold recycled mixes (CRME) by varying the passage rate of 4.75 mm sieve holes. After age-conditioning of the CRME samples for different times (0, 5, and 10 days), it was observed that the low-temperature cracking resistance deteriorated progressively over time, regardless of the type of CRME investigated. Zhou et al. [94] attempted to address the negative effects of RAP on long-term thermal crack resistance by adding a rejuvenator to the RAP mixture, but obtained unfavorable results.

Monu et al. [101] investigated the long-term performance effects of adding WMA additives to RAP blends. After long-term aging in the laboratory, it was found that densely graded asphalt mixes containing 35% RAP and WMA additives showed greater improvements in water resistance, durability, and rutting resistance than mixes without WMA additives.

Puppala [102] focused on the long-term performance of RAP applied to ACP subgrade. Based on water durability, leaching, and mineralogical testing of untreated and treated RAP subgrade mixtures, it was concluded that good long-term performance was best achieved with 75% RAP in the subgrade. Avirneni et al. [103] conducted a similar study, but with the addition of stabilizers and activators to the RAP subgrade mixtures. The water durability met local requirements, while the long-term unconfined compressive strength did not show any signs of reduction over time. Elsewhere, Isola et al. [104] used cement to treat a RAP base and foundation mix containing 70% RAP. After 15 months of monitoring, it was found that the sections with cement-treated RAP mixes outperformed those with conventional cement-treated non-RAP mixes in terms of stiffness.

**Figure 7:**
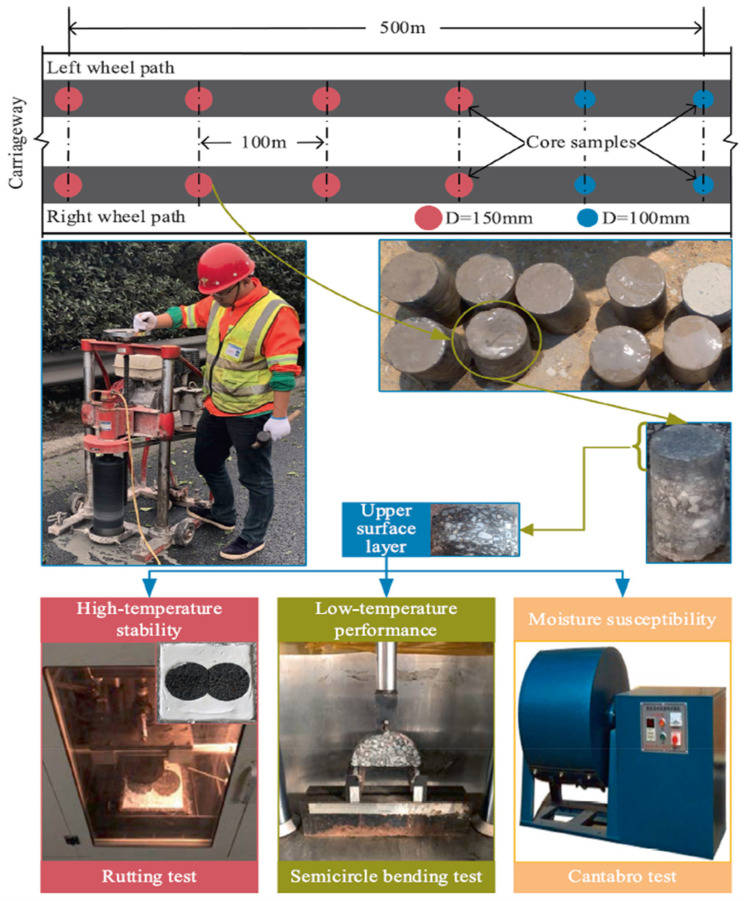
Core sample extraction for laboratory tests (Reprinted with permission from Pan et al. [105]. 2021, Elsevier).

More recently, Pan et al. [105] investigated the long-term performance of ACPs maintained by hot in-place recycling (HIR) in Jiangsu Province, China. HIR is a construction process that fully reuses RAP, a non-renewable resource, requiring only 10–20% virgin material and a limited amount of rejuvenator [106]. Figure 7 shows sample cores taken from different sections of the pavement after 5 years of HIR use, and the laboratory tests carried out.

Based on the road performance tests shown in Figure 7, Pan et al. [105] determined that the HIR technology improved high-temperature stability, maintained acceptable low-temperature and moisture-sensitivity performance, and could extend the life of the pavement by at least 5 years.

The above-mentioned studies have suggested that RAP-ACP, if designed and constructed correctly, can have similar or better long-term performance as compared to virgin ACP.

## 5. Reflections from the Literature Review and Perspectives for Future Research

Despite the current efforts in ACP recycling, the quantity of RAP produced has not been fully utilized, and as a result, more innovative recycling alternatives are required. Already, there are claims that 100% RAP can be successfully used in the pavement asphalt layers [11], and that RAP usage can be extended even to the pavement foundation layers [47,51,53,54,55]. For the latter, stabilization of the granular materials using techniques including mechanical, chemical, and cementitious binders can allow for more RAP usage. However, the cost of the stabilizing agents and the special expertise required for its implementation could be a hindrance towards achieving popularity equal with the application of RAP in asphalt layers. In addition to these, more alternative ways have also been considered for maximizing RAP application in ACP, including the use of RAP in PPM treatments and RAP multiple recycling.

The literature on RAP-PPM and R^n^AP are very scarce, and despite the promising results on their durability and performance, some important aspects need to be adequately addressed. Nevertheless, they are essential to maximize and extend the beneficial usage in asphalt pavement industry. This section covers important topics that have not been addressed so far for the future development of RAP-PPM and R^n^AP, as well as the understanding of the long-term performance behavior of RAP-ACP.

### 5.1. Potential Challenges and Future Research Needs for RAP-PPM

The application of RAP in PPM treatment mixtures is relatively new and is not yet widely studied. As evident from Figure 2, only limited research has been conducted so far, and from what is known, Table 1 identifies the biggest challenge as the design of an RAP-PPM mix that is workable and has positive cohesion test results.

The poor cohesion results commonly reported in the various studies suggest a lack of proper bonding between the RAP materials and virgin materials during the RAP-PPM production. Several factors such as RAP characteristics (gradation, amount, source), additives, and asphalt emulsion type and amount can influence both the bonding characteristics and workability of the RAP-PPM mix. For example, using higher RAP content in PPM treatments would require more water and less additive, which would retard the setting of the mixture and cause bonding problems [60]. However, reducing the RAP amount does not necessarily mean the problem will be solved. Typically, the use of more RAP fines is recommended in PPM treatments. The more material that passes through the smallest sieve, the greater the surface area of the mixture and the more emulsion is required to coat all the dispersed particles. In addition, the more angular the particle shape and the rougher the surface texture, the higher the emulsion content required for workability. Both of these factors influence the optimum emulsification requirements for RAP, as the fines adhering to the larger particles reduce the total mixing surface area but also make the particles more irregular in shape, offsetting the effect to some extent. It is not well understood what contribution the bitumen-coated particles in the RAP make to the mix. RAP asphalt will age to varying degrees during use, and cannot be expected to perform as well as virgin asphalt or newly produced asphalt emulsions. More research is required on how RAP asphalt interacts with emulsions and other treatments.

Mix design modifications to accommodate RAP in PPM treatments should establish volumetric requirements from standard performance-based criteria of the proposed PPM treatment. Once a mix design has been produced, the RAP material should be processed immediately to minimize variability and limit the effects of factors such as bonding and workability.

Another gap in the literature is the lack of information on the long-term performance of RAP-PPM mixtures. The expected service-life of virgin PPM treatments is 7 to 10 years. Future research should document whether RAP treatments meet or exceed this service life. Verification is also needed to show viability of RAP-PPM treatments over a wide range of climates. The durability of trial mix designs and completed projects must be documented, which will help to properly refine the specification and mix design of a proposed PPM treatment for the successful incorporation of RAP.

### 5.2. Potential Challenges and Future Research Needs for R^n^AP

Existing research on repeated recycling of RAP is limited to laboratory-based studies and assesses performance at a macro level, which is insufficient to facilitate practical applications. It is imperative that chemical and microstructural characterization of R^n^AP blends and field performance investigations of actual ACP containing R^n^AP mixtures are carried out and the results are correlated with laboratory results to provide a comprehensive understanding of the repeatable recycling of ACP.

#### 5.2.1. Molecular and Chemical Characterization

First, a comprehensive understanding on the performance mechanism of R^n^AP is still lacking due to the insufficient research on this subject. This is evident by some conflicting findings in the literature. For example, some researchers [84] indicated that the viscosity of RAP binder is not affected after multiple recycling, while others [86] reported that the viscosity of RAP binder is significantly increased after several recycling processes. It is known that low viscosity binders have higher molecular movement and faster diffusion of oxygen than highly viscous binders [92] Therefore, by characterizing the chemical changes in the binders as a result of oxidation after multiple recycling, useful information would be provided to help understand the viscosity changes as well as other performance properties of R^n^AP binder.

#### 5.2.2. Microstructural Characterization

So far, the existing studies on the performance properties of R^n^AP have only been at the macroscale level. Based on the conclusions arrived by several researchers [81,82,83,85], the LVE properties of RAP binder is not significantly affected by multiple recycling, and when compared with virgin asphalt binder, R^n^AP at a maximum dosage of 40% has better rutting and fatigue resistance. However, such results can be further verified and well-supported by constructive microstructural characterization of the R^n^AP binder, which is still lacking in the literature. Popular microstructural characterization techniques such as the atomic force microscopy (AFM) method is capable of detecting topographic (height) and LVE (e.g., stiffness and adhesion) features on surfaces. The characteristics (e.g., size) of the so-called “bee structure” on the surface of bitumen is a consequence of a number of factors (e.g., chemical composition, intermolecular interactions, colloidal interactions, and binder viscosity), while the phase images can provide indication of mechanical properties of the surface features [92]

#### 5.2.3. R^n^AP Pavement Field Performance

Another major concern is the lack of data from actual pavements containing R^n^AP mixtures. The current knowledge on R^n^AP performance is entirely based on laboratory-prepared binders and mixtures. Conventional laboratory aging protocols were used in previous studies to simulate R^n^AP and laboratory test methods were used to investigate their performances. It is imperative to carry out field performance investigations on actual ACPs containing R^n^AP mixtures and the results correlated with laboratory results in order to provide a comprehensive understanding of the multiple recycling of ACPs.

### 5.3. RAP-ACP Long-Term Performance Research

In almost all the investigations conducted so far, data from FHWA’s Long-Term Pavement Performance (LTPP) program were utilized, while a few others were based on laboratory simulation. Despite the existing studies showing promising results in the LTTP program data, the analysis results can vary due to several reasons such as incomplete data set or uncertainty in the data. Moreover, although LTPP has been collecting pavement distress data, including various types of cracking and rutting, for many years, there has not been a standard method to calculate an overall pavement distress index on the basis of the collected distress data [96]. It is therefore necessary to continue developing more reliable analysis results that are based on an extensive and comprehensive data of all factors influencing the long-term performance of RAP-ACP. Sound performance models established on real-world data for pavement life prediction are necessary for accurate calculation and thus, evaluation of RAP’s engineering sustainability benefits.

## 6. Summary and Conclusions

For the past decades, RAP recycling has been a major drive towards achieving greater sustainability in the asphalt pavement industry. Due to the significant cost and environmental benefits of RAP, various innovative ways have been developed on how to fully exploit this material in ACP, such as 100% replacement of new materials with RAP, improved construction techniques (e.g., HIR and CIR recycling methods), and application in pavement foundation layers. In addition to these, recent years of research have introduced the possibility that RAP can be utilized in PPM treatment mixtures and that RAP-ACP can be recycled several times without significantly affecting pavement performance. Certainly, these two approaches are additional ways for maximizing and extending the beneficial reuse of RAP. However, their widespread implementation is particularly hindered because of lack of sufficient research to provide a comprehensive understanding about various aspects such as their mixture formulation, mechanical and functional performance, durability, and cost implications. This paper systematically reviews the various studies reporting on RAP-PPM and R^n^AP technologies and evaluates the existing knowledge, identifies the major challenges, and highlights the gaps and future research needs. The long-term behavior of traditional RAP-ACP was also reviewed based on influencing factors and field performance data. Based on this study, the following conclusions are drawn.

1. The use of RAP in PPM treatment mixtures is an innovative concept to help maximize its application in asphalt pavements, thereby reducing the amount of remaining RAP. It is possible to slightly adjust existing mixture designs for THMAO, micro-surfacing, chip seals, and slurry seals in order to accommodate RAP materials. Fractionation of RAP produces more fines, and so, to make the most of this resource, more RAP fines should be used in the mix designs of PPM treatments. Performance-wise, the literature reported similar or better performance of RAP-PPM than PPM containing VA.
2. Bonding and workability are the main challenges that must be addressed in order to fully advance RAP-PPM technology. It is necessary to understand how several factors such as RAP characteristics (gradation, amount, source), additives, emulsion type, and amount could help improve the bonding issues and workability of the RAP-PPM mix components and correlate the results with mixture performance.
3. The amount of research on RAP-PPM is very limited. Most studies only focus on the modification of PPM treatment mix design to incorporate RAP, and how RAP inclusion affects the mechanical performance of PPM treatment mixtures. Still little or nothing is known about the functional properties, long-term performance, or cost-effectiveness of RAP-PPM treatment mixtures, which should be considered when aiming for sustainability.
4. RAP can be recycled several times, which can extend the life of the materials until they can no longer be used. However, if the repeatedly recycled RAP materials are not properly incorporated into a new mix, various performance characteristics such as stiffness or durability properties can be adversely affected, leading to premature pavement failure and, ultimately, more frequent pavement repairs and rehabilitation.
5. Research on R^n^AP mixtures have only been limited to the macro level. Detailed understanding of how multiple recycling can influence the chemical and molecular structure of fresh asphalt mixtures is vital to the performance improvements of R^n^AP mixtures.
6. Although the recycling of RAP can reduce costs, there are still some concerns about ACP containing RAP, particularly in the load-bearing layers. A major concern is its durability and long-term performance under various exposure conditions. Knowledge of the long-term performance of RAP-ACP is important, and without it, confidence in its widespread implementation may be reduced. Available literature suggests that RAP has a negative impact on long-term cracking performance, but performs similarly or better than ACP without RAP in terms of rutting resistance, raveling, and ride quality.

## Figures and Tables

**Table 1:** Summary results on RAP-PPM mixture performance properties.

PPM Treatment	RAP Amount (%)	Rejuvenator	Rutting Resistance	Low-Temperature Cracking	Fatigue Resistance	Moisture Susceptibility	Workability	Skid Resistance	Cohesion	Ref.
THMAO	40	None	+			+	−			[42]
Chip seal	20	None	+	O						[57]
Microsurfacing	50 & 100	None	+			O			−	[58]
Microsurfacing	50 & 100	None	+			+				[59]
Microsurfacing	69	None	+			+			−	[60]
Slurry seal	87.5	None	+			+			−	[61]
Microsurfacing	20–40	Chemical				+		+	−	[62]
THMAO	40	Biological	+	+	+					[63]

+ positive effect; − negative effect; O insignificant effect.

**Table 2:** Effect of RAP on long-term performance.

Ref (s).	Performance Investigated		Long-Term Performance Duration (Years)	RAP Amount Utilized (%)	Performance Indicator	Effects as Compared to Virgin ACP
[89]	Cracking of various types	Fatigue cracking	0–12	30	Crack initiation time	Worse
[90]			Long-term oven aging (LTOA) at 85 °C for 5 days		DCSE_f_ and Plateau Value	
[91]			10–15		Fatigue cracking area (m^2^)	
[92]			LTOA at 85 °C for 5 days	40	Fatigue life	
[93]		Transverse cracking	16	35	Transverse cracking length	
[91]			10–15	30	Number of cracks/sections	
[94]		Low-temperature cracking	LTOA at 85 °C for 5 days	15, 30, 40, 50	60 s stiffness and m-value	
[91]		Longitudinal cracking	10–15	30	Longitudinal cracking length (m)	
		Block cracking			Block cracking area (m^2^)	Similar
[95]	Rutting		8–17	30	Deflection	Similar
[93]			16	35	Rut depth (mm)	Worse
[91]			10–15	30		Similar
[91]	Raveling		10–15	30	Raveling area (m^2^)	Similar
[93]	Ride quality		16	35	International Roughness index (IRI)	
[96,97]			10–15	30		
[91]						
